# A Janus‐Like Bio‐Inspired Strategy for 3D‐Printed Bimetallic Metamaterials with Excellent Thermal‐Protection and Load Bearing Capacity

**DOI:** 10.1002/advs.202524116

**Published:** 2026-02-05

**Authors:** Zhicheng Dong, Wei Cheng, Yu He, Ben Jia, Xiaopeng Wan, Heyuan Huang

**Affiliations:** ^1^ School of Aeronautics, Northwestern Polytechnical University Xi'an China; ^2^ School of Civil Aviation Northwestern Polytechnical University Xi'an China; ^3^ Shaanxi Provincial Science and Technology Industry Innovation Center for “Aircraft Composite Material Structure Design and Application” Xi'an China; ^4^ National Key Laboratory of Aircraft Configuration Design Xi'an China

**Keywords:** 3D printing, bimetallic metamaterials, janus‐like bio‐inspired strategy, numerical simulations, thermal‐mechanical performance

## Abstract

Functional structures that combine thermal protection with load‐bearing capabilities represent an effective solution to hypersonic thermal‐protection challenges. Here, we propose a Janus‐like bio‐inspired strategy for integrally 3D‐printed bimetallic metamaterials. Inspired by shell bilayers, a heat‐resistant AlSiFeMnNiMg alloy and a SiC‐reinforced AlSi10Mg are arranged as an architected pair and fabricated via dual‐hopper selective laser melting, with SiC volume fractions of 0, 4, and 8 vol%. In situ SEM tensile tests at 25°C and 250°C show that damage is confined to a narrow transition zone. Once one side softens, the bimetallic architecture redirects load to the other, forming non‐percolating high‐stress paths and stabilizing the plateau response. Quasi‐static compression of Gyroid TPMS lattices with different SiC contents maps the composition‐temperature space. Across temperatures, structures with 4 vol% SiC improve specific energy absorption by 11.72% and 18.67% in room temperature and by 10.28% and 18.8% in 250°C, achieving synergistic mechanical improvement and a stable energy‐absorbing plateau under extreme environments. Relative to 0 and 8 vol%, where modulus mismatch precipitates premature localized collapse, 4 vol% SiC promotes a distributed shear‐band network that delays failure and elevates load capacity. This work provides a practical pathway toward thermally protective and load‐bearing integrated components for aerospace applications.

## Graphical abstract

A Janus‐like bio‐inspired strategy is proposed for integrally 3D‐printed bimetallic metamaterials. Inspired by shell bilayers, a heat‐resistant AlSiFeMnNiMg alloy and a SiC‐reinforced AlSi10Mg with different SiC volume fractions are arranged as an architected pair. Across temperatures, structures with 4 vol% SiC achieve greatest synergistic mechanical improvement under extreme environments, opening a design space for thermally protective and load‐bearing integrated components.

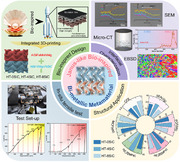

## 1 Introduction

Hypersonic vehicles operate within extreme aero‐thermal environments where steep thermal gradients coexist with complex multiaxial mechanical loads induced by aerodynamics, vibration, and impact [1, 2]. Under such coupled thermo‐mechanical conditions, structural systems must simultaneously provide reliable thermal protection, retain load‐bearing stability at elevated temperatures, and dissipate energy against transient events [3]. This intrinsic multi‐objective conflict motivates the development of functional structures that combine thermal protection with load‐bearing capabilities, representing an effective solution to such hypersonic thermal‐mechanical challenges [4].

Bio‐inspired structural design offers an effective framework for this integration by translating the natural division of labor into engineered architectures [5]. In particular, layered biological systems, such as mollusk shells, employ a hard exterior to resist external attack and a compliant interior to dissipate energy, providing a synergistic balance between protection and mechanical robustness [6]. Building on such Janus‐like bi‐layered archetypes, bimetallic architectures have emerged as a promising strategy to reconcile competing requirements by combining two metals with complementary properties [7, 8, 9, 10]. Crucially, under high‐temperature service, the appropriate spatial arrangement of a heat‐resistant alloy with a conventional alloy enables a graded coefficient of thermal expansion, mitigating thermally induced internal stresses, and thereby improving thermal‐fatigue resistance and durability [11, 12]. Notably, practical thermal protection systems (TPS) already implement this principle through multi‐material functional partitioning. Classic TPS concepts, exemplified by the Space Shuttle, typically employ an external thermal layer and an internal load‐bearing substructure [3]. Yet, such architectures are commonly realized through discrete stacking and secondary assembly using adhesives or fasteners [13, 14]. These interfaces introduce parasitic mass and geometric discontinuities, which may compromise reliability under cyclic thermo‐mechanical fatigue. These limitations underscore the need for advanced manufacturing approaches capable of fabricating complex, integrated multi‐material architectures with controlled interfaces [15].

Selective laser melting (SLM), a powder‐bed fusion additive manufacturing technology, offers a bottom‐up route to high‐quality, integrated bimetallic fabrication via layer‐wise building and near‐net‐shape capability [16, 17, 18, 19]. For example, Zhao et al. fabricated 316L/18Ni300 multi‐material components by SLM, leveraging the complementary toughness of 316L and the ultra‐high strength of 18Ni300 [20]. Nevertheless, bi‐material additive manufacturing has predominantly focused on steels, copper or titanium alloys, whereas aluminum‐alloy‐based bi‐material designs remain limited despite their importance for lightweight aerospace structures owing to the favorable strength‐to‐weight ratio and lightweight property [21, 22].

Moreover, commonly used printable aluminum alloys, such as AlSi10Mg, suffer intrinsic limitations in high‐temperature mechanical performance and fracture toughness [19, 23]. Specifically, above approximately 200°C, pronounced thermal softening renders these alloys to simultaneously deliver thermal protection and structural load‐bearing under coupled thermo‐mechanical fields, resulting in over 20% strength loss [24, 25, 26]. To address this, doping‐based strengthening strategies have been widely explored [27]. Research indicates that adding Fe, Ni, and Mn to Al‐Si alloys promotes the precipitation of thermally stable Al_5_(FeNi) and Al_6_Mn phases, thereby enhancing structural load‐bearing capacity at elevated temperatures [28]. Complementarily, aluminum‐based silicon carbide (Al/SiC) utilizes the difference in coefficient of thermal expansion and stiffness between SiC particles and the aluminum matrix [29, 30]. This mismatch introduces residual stress and multiplies dislocation density, resulting in high stiffness, and deformation resistance. Studies show that doping with 10 vol% SiC particles can increase the ultimate strength of the matrix by approximately 35.0%. Thus, incorporating SiC particles yields a composite with high strength, stiffness, and dimensional stability suitable for load‐bearing connections [31].

Importantly, SLM also minimizes secondary damage associated with conventional machining and excels at integrating multiple materials within complex geometries [32, 33].To fully exploit Janus‐like bi‐material strategies in high‐temperature energy‐absorption scenarios, architected lattice metamaterials serve as effective structural carriers due to their lightweight yet high‐strength characteristics. Ding et al. showed that Ti/Nb bi‐material lattices with Gyroid architectures exhibit robust interfacial bonding and superior overall strength [34]. This highlights the promise of combining Janus‐like material partitioning with Triply Periodic Minimal Surface (TPMS) metamaterials for an integrated thermal protection‐load bearing solution under hypersonic constraints.

Against this background, we select AlSiFeMnNiMg and AlSi10Mg‐xSiC (x = 0, 4, and 8 vol%) as the constituents for an SLM‐fabricated bimetallic architecture. Our selection of the AlSiFeMnNiMg alloy is motivated by its refined microstructure and ability to retain strength at elevated temperatures. It is paired with SiC‐reinforced AlSi10Mg, a high‐specific‐strength aluminum‐matrix composite that is widely used for aerospace‐relevant lightweight applications. We undertake a systematic, multiscale investigation of microstructural evolution, and interfacial chemistry in AlSiFeMnNiMg/AlSi10Mg‐xSiC bimetals, with emphasis on interfacial bonding. Along the build direction, we perform Micro‐CT, SEM, and in situ SEM tensile test to clarify crack initiation and growth. We compare microstructures and mechanical behavior across SiC volume fractions to quantify how particle content governs interfacial control and property tuning. Finally, we extend our study to heterogeneous TPMS lattices produced by SLM, contrasting their microstructure and mechanical response in complex architectures. Collectively, these experiments and mechanistic analyses furnish design guidance for particle‐reinforced interfacial engineering and multiscale performance optimization in bimetallic lattices, validating the technical feasibility and application potential of the present dual‐hopper SLM bimetal strategy for next‐generation high‐end manufacturing.

## 2 Results and Discussion

### 2.1 Bi‐Metallic Design Criteria and 3D Printing Technology

**FIGURE 1:**
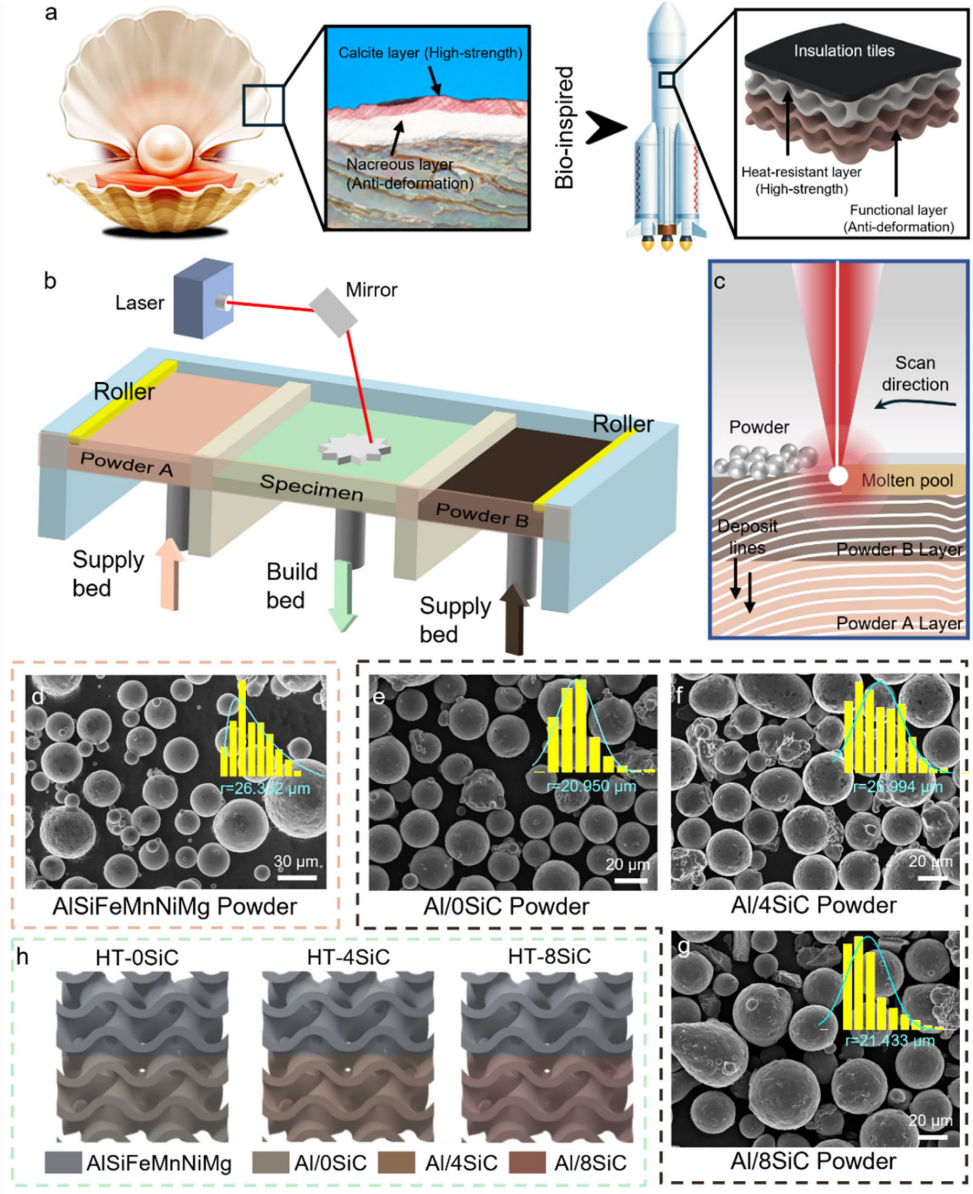
Bio‐inspired bi‐metallic structures. (a) Bilayer architectures in shells and thermal protection systems. (b) Schematic of the customized dual‐hopper SLM 3D‐printing process. (c) Illustration in terms of the printing configuration. (d–g) Micrographs of (d) AlSiFeMnNiMg powder, (e) Al/0SiC powder, (f) Al/4SiC powder, and (g) Al/8SiC powder. (h) Bi‐metallic TPMS structures with the unit‐cell size of 5 mm×5 mm×5 mm. A 6 × 6 × 6 cell tessellation yielded lattice blocks of 30 mm × 30 mm × 30 mm.

In nature, shells achieve outstanding mechanical efficiency at low weight by combining a hard exterior that resists external impact with a tough, energy‐dissipative interior that suppresses deformation [35]. This division‐of‐function paradigm offers a fresh blueprint for lightweight metallic structures in spacecraft (Figure 1a) [36]. Inspired by this concept, we designed and monolithically fabricated a 3D‐printed Janus‐like bimetallic architecture. In our strategy, a high‐strength, heat‐resistant AlSiFeMnNiMg alloy serves as the structural heat‐resistant layer, imparting thermal protection, and load‐bearing capability. An AlSi10Mg doped with SiC at tailored fractions as a functional layer enhances overall stiffness and deformation resistance. Specifically, the AlSiFeMnNiMg alloy incorporates transition‐metal elements that promote the formation of thermally stable Al_5_(FeNi) and Al_6_Mn phases during processing. These heat‐resistant phases pin grain boundaries, thereby markedly improving microstructural integrity and load‐retention capability at elevated temperatures. For the load‐bearing functional layer, we selected a SiC‐reinforced AlSi10Mg composite that has been widely adopted in aerospace applications. This composite leverages the mismatch in coefficient of thermal expansion and stiffness between SiC particles and the Al matrix to introduce additional residual stresses and increase dislocation density during manufacturing and deformation, resulting in high stiffness, and strong resistance to deformation. Importantly, when introducing ceramic reinforcement, we explicitly accounted for a key design criterion that excessive modulus mismatch may induce interfacial brittleness and elevate failure risk [37]. Prior studies have shown that overly high particle content and aggregation can compromise the strength‐toughness balance [38]. Therefore, we set the SiC volume fraction as a graded variable at 0, 4, and 8 vol% to quantitatively evaluate how particle content governs interfacial microstructural continuity, cross‐interface load‐transfer efficiency, and failure modes within the composition space, ultimately identifying the optimal strength‐toughness matching strategy.

Meanwhile, achieving superior mechanical performance, particularly high energy‐absorption capability, also critically depends on selecting an architected topology with strong load‐carrying capacity for the proposed Janus‐like bio‐inspired aluminum‐based bimetal [39, 40]. Here, we adopt the Gyroid‐type triply periodic minimal surface (TPMS) rather than conventional strut‐based lattices such as BCC or FCC. First, the gyroid features a continuous surface with smoothly varying mean curvature [41]. Compared with strut lattices that suffer from pronounced nodal stress concentrations, TPMS architectures more effectively alleviate localized stress amplification at the bimetallic interface arising from thermo‐mechanical mismatch, thereby reducing the risk of interfacial debonding [42]. In addition, the distribution and high connectivity of the gyroid promote efficient and stable energy absorption under complex loading [43].

Figure 1b,c summarizes the selective laser melting (SLM) workflow used to integrally fabricate the AlSiFeMnNiMg/AlSi10Mg‐xSiC (denoted as HT‐xSiC) bimetallic specimens and shows representative powder morphologies at different material states. Gas‐atomized AlSiFeMnNiMg powder and spherical AlSi10Mg powder premixed with SiC particles at designed volume fractions x = 0, 4, and 8 vol% served as the feedstocks. SEM observations (Figure 1d–g) indicate that AlSiFeMnNiMg (denoted as HT) exhibits high sphericity and good flowability, whereas the addition of SiC increases surface roughness and introduces some irregular particles in the Al‐based composite, which may influence powder‐bed packing density and melt‐pool wetting behavior. For the architected specimens, a triply periodic minimal surface (TPMS) lattice based on the Gyroid unit cell was adopted [44]. The unit‐cell size was 5 mm × 5 mm × 5 mm. A 6 × 6 × 6 cell tessellation yielded lattice blocks of 30 mm × 30 mm × 30 mm (Figure 1h). The lattice volume fraction was defined as the ratio of the solid volume to the total envelope volume and was computed in the CAD environment, which was set as 35% in this research. Three material pairings were implemented for the bimetal TPMS structures:(1) AlSiFeMnNiMg/ AlSi10Mg‐0SiC (denoted as HT‐0SiC); (2) AlSiFeMnNiMg/AlSi10Mg‐4SiC (denoted as HT‐4SiC); (3) AlSiFeMnNiMg/AlSi10Mg‐8SiC (denoted as HT‐8SiC). Printed specimen can be found in Figure S1. With increasing SiC fraction, the bimetallic lattices display a more pronounced color contrast and a sharper transition interface, being consistent with the intended material partitioning.

### 2.2 Quality Inspection and Microstructure Characteristics

**FIGURE 2:**
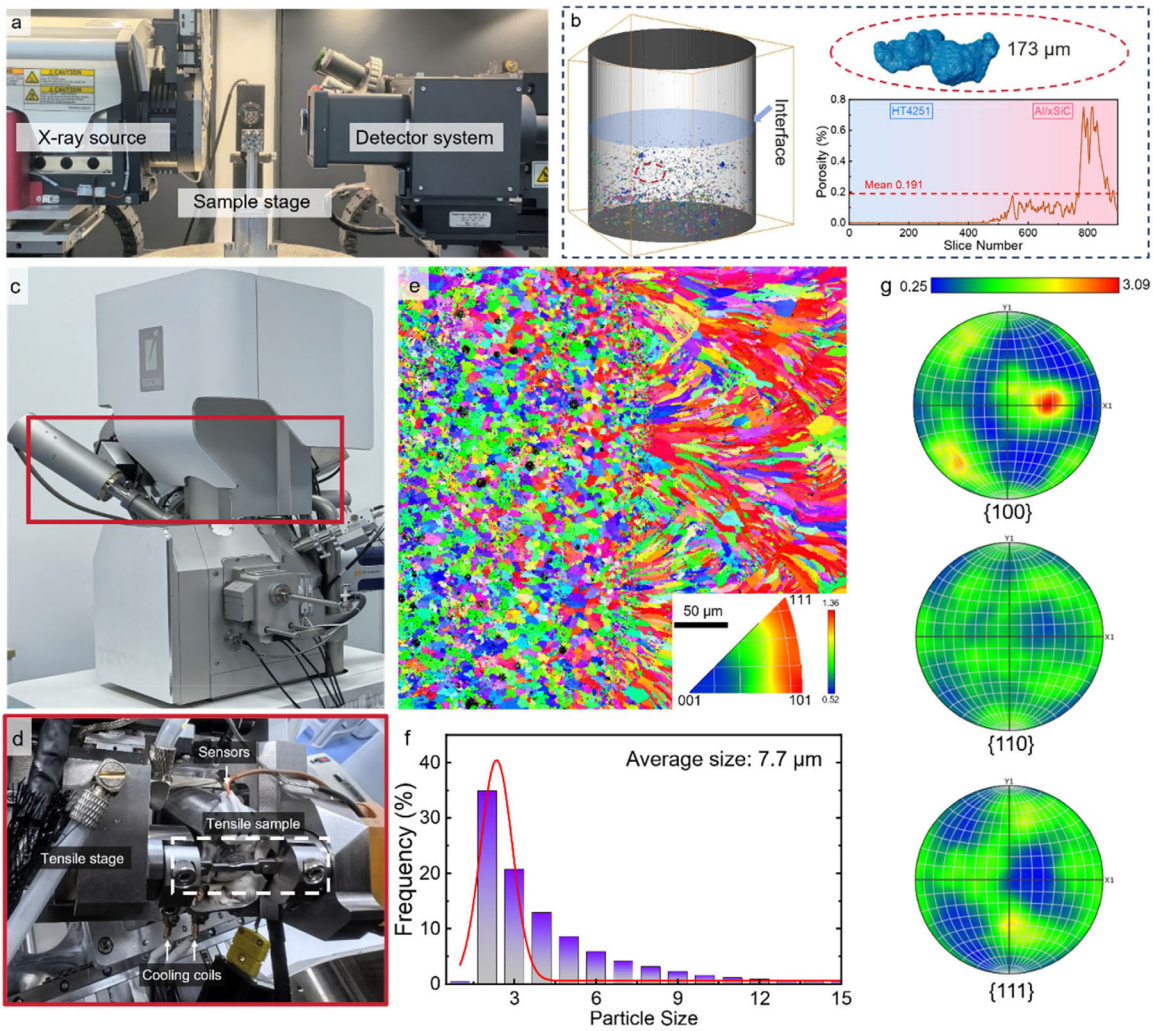
Multiscale characterization and in situ setup for the bio‐inspired bimetallic alloy. (a) Micro‐CT system used to survey internal defects across the printed bimetal, showing X‐ray source, sample stage and detector. (b) 3D reconstruction of the HT‐8SiC cylinder with the interface indicated; pores are segmented in blue and remain isolated without cross‐interface connectivity (inset: largest pore about 173 µm). (c) In situ SEM tensile configuration used to probe interfacial deformation. (d) Detailed view highlighting the miniature tensile stage, grips, sensors and cooling coils enabling stable loading and imaging. (e) EBSD inverse‐pole‐figure (IPF) map across the interface prior to deformation: the AlSi10Mg‐xSiC side exhibits fine equiaxed grains and weak texture, whereas the AlSiFeMnNiMg side shows feather‐like dendritic morphology with pronounced orientation. (f) Grain‐size distribution (histogram) with a log‐normal fit (red). (g) Pole figures corresponding to the EBSD map.

Pores diminish the stress‐effective load‐bearing area and markedly degrade mechanical properties, most notably for strength and elongation [45]. The schematic of the scanning setup and the pore‐reconstruction results for HT‐8SiC components (Figure 2a,b) show that the printed bimetal forms a dense, compositionally partitioned interface that predisposes the system to cooperative load sharing under deformation. Numerous pores are observed in the Al/8SiC side, whereas pore populations are substantially lower in the AlSiFeMnNiMg side. The maximum equivalent pore diameter in Al/8SiC is about 173 µm. Across the metallurgical bond, the porosity profile exhibits only a narrow transition with no continuous porous band. Statistical pore analysis yields an average porosity of 0.191%, a maximum of 0.68%, and a minimum of 0.012% for the Al/8SiC component.

Figure 2c,d illustrates the in situ tensile configuration, highlighting specimen positioning and alignment. Prior to deformation, the bimetal exhibits distinct initial microstructures on the two sides of the interface. On the left, the AlSi10Mg‐xSiC region comprises equiaxed, recrystallized grains ∼3–10 µm in size with a uniform grey contrast. Fine SiC particles are homogeneously dispersed without obvious clustering. On the right, the AlSiFeMnNiMg region exhibits feather‐like dendritic features with a preferred <001>/<110> orientation, characterized by long axes >100 µm and lamellar widths of ∼10–15 µm. The EBSD inverse pole figure (IPF) map (Figure 2e) corroborates these observations with the AlSi10Mg–xSiC side showing a broad color distribution indicative of very weak texture, whereas the AlSiFeMnNiMg side presents banded, near single‐color domains reflecting a heat‐flow‐induced preferred orientation. The corresponding grain‐size histogram (Figure 2f) shows an average equivalent diameter of about 7.7 µm. The finely dispersed SiC particles are expected to strengthen the alloy through grain‐boundary strengthening, while the few coarse particles may act as potential void/crack nucleation sites during subsequent tensile loading. To quantify intragranular strain heterogeneity prior to loading, the kernel average misorientation (KAM) was evaluated. As shown in the Figure S2, the mean KAM in the AlSi10Mg‐8SiC region is ∼0.9°, whereas the AlSiFeMnNiMg side averages ∼0.3°. These microstructure–interface attributes jointly govern load‐path reconfiguration during deformation: when the more compliant side first undergoes local buckling or softening, the opposite side assumes and redistributes load along TPMS nodes and struts. Consequently, stress accumulates as discrete clusters across neighboring unit cells rather than forming a through‐thickness percolating band. The resulting reduction in stress connectivity suppresses the coalescence of interfacial damage into long‐range shear bands, retards crack propagation, and macroscopically manifests as a more stable stress plateau and delayed collapse.

**FIGURE 3:**
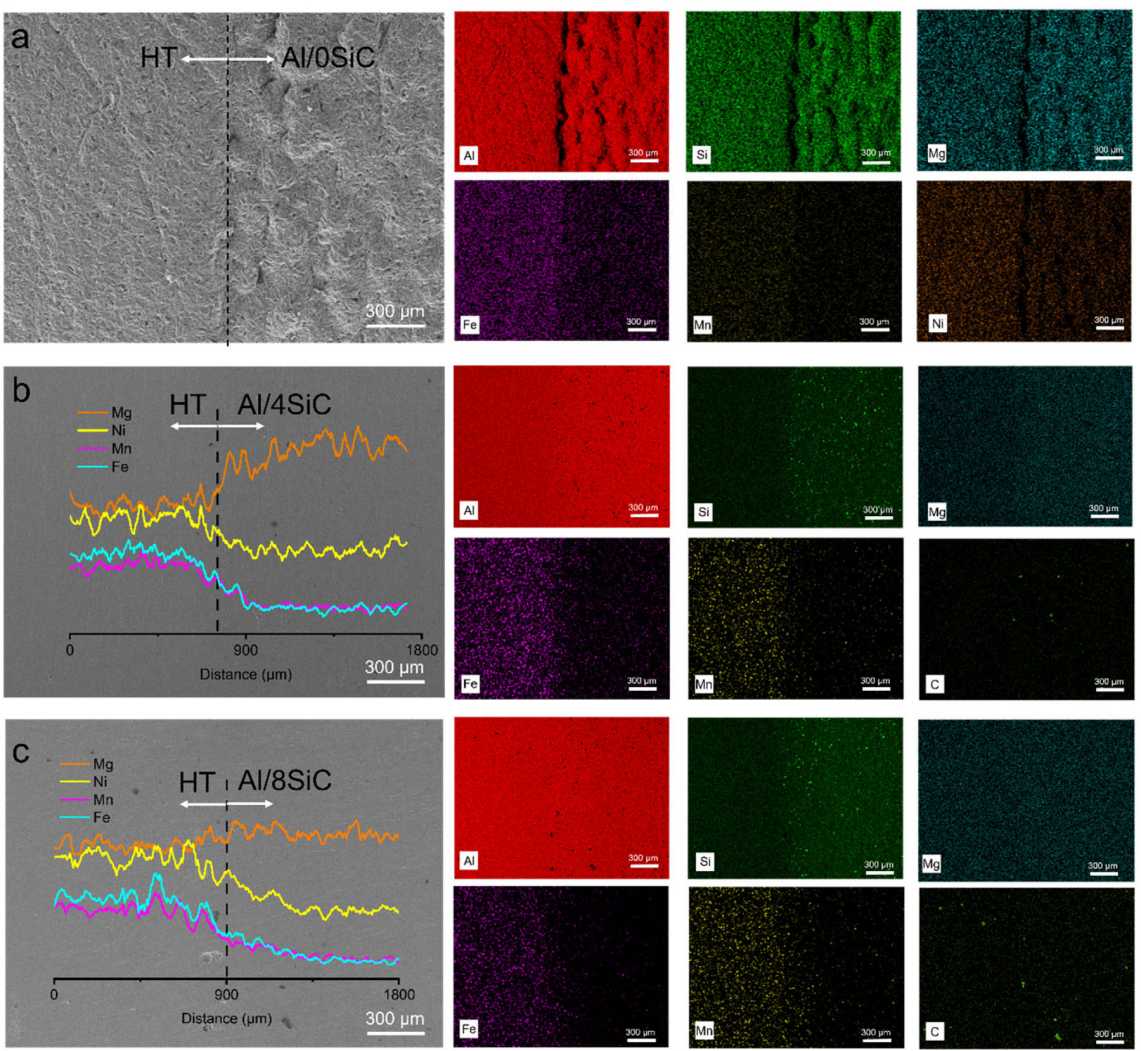
Fine microstructures and element distributions in bioinspired HT‐xSiC bimetallic structures. (a) HT‐0SiC, (b) HT‐4SiC, (c) HT‐8SiC.

Figure 3 presents SEM morphologies and elemental maps of the AlSiFeMnNiMg/Al‐xSiC (x = 0, 4, and 8 vol%) heterogeneous interfaces, providing direct evidence of compositional continuity and metallurgical bonding. Aluminum and silicon remain continuous and nearly uniform across the field of view, indicating Al─Si matrices on both sides of the interface. By contrast, Fe and Mn are clearly enriched on the AlSiFeMnNiMg side and decay to background levels within a short distance across the interface, demonstrating only minimal dilution and cross‐boundary diffusion during dual‐hopper SLM powder switching and scan transitions. Meanwhile, carbon is confined to the Al‐xSiC side and increases in areal coverage as the SiC fraction rises from 0 to 8 vol%. No significant C enrichment is detected on the AlSiFeMnNiMg side, implying that SiC particles neither migrated across the boundary nor underwent noticeable decomposition. No unmeted defects or continuous porosity bands are observed in any sample, attesting to reliable metallurgical bonding. In contrast to common multi‐material additive manufacturing issues, our dual‐channel powder feeding and spatial material‐partitioning strategy effectively compresses the interfacial transition width and suppresses the formation of continuous intermediate phases. In tandem with coordinated optimization of powder flowability and the process energy‐density window, this approach prevents continuous porous or unmelted regions near the interface, thereby enhancing interfacial structural integrity and reproducibility. Taken together, the SEM‐EDS evidence substantiates the proposed heterogeneous additive strategy, which preserves robust metallurgical bonding while enabling customized control over ceramic‐phase content and spatial distribution, establishing a validated design and manufacturing basis for lightweight energy‐absorbing, load‐bearing components in high‐temperature environments [46, 47].

### 2.3 In Situ Tensile Tests at Elevated Temperatures

**FIGURE 4:**
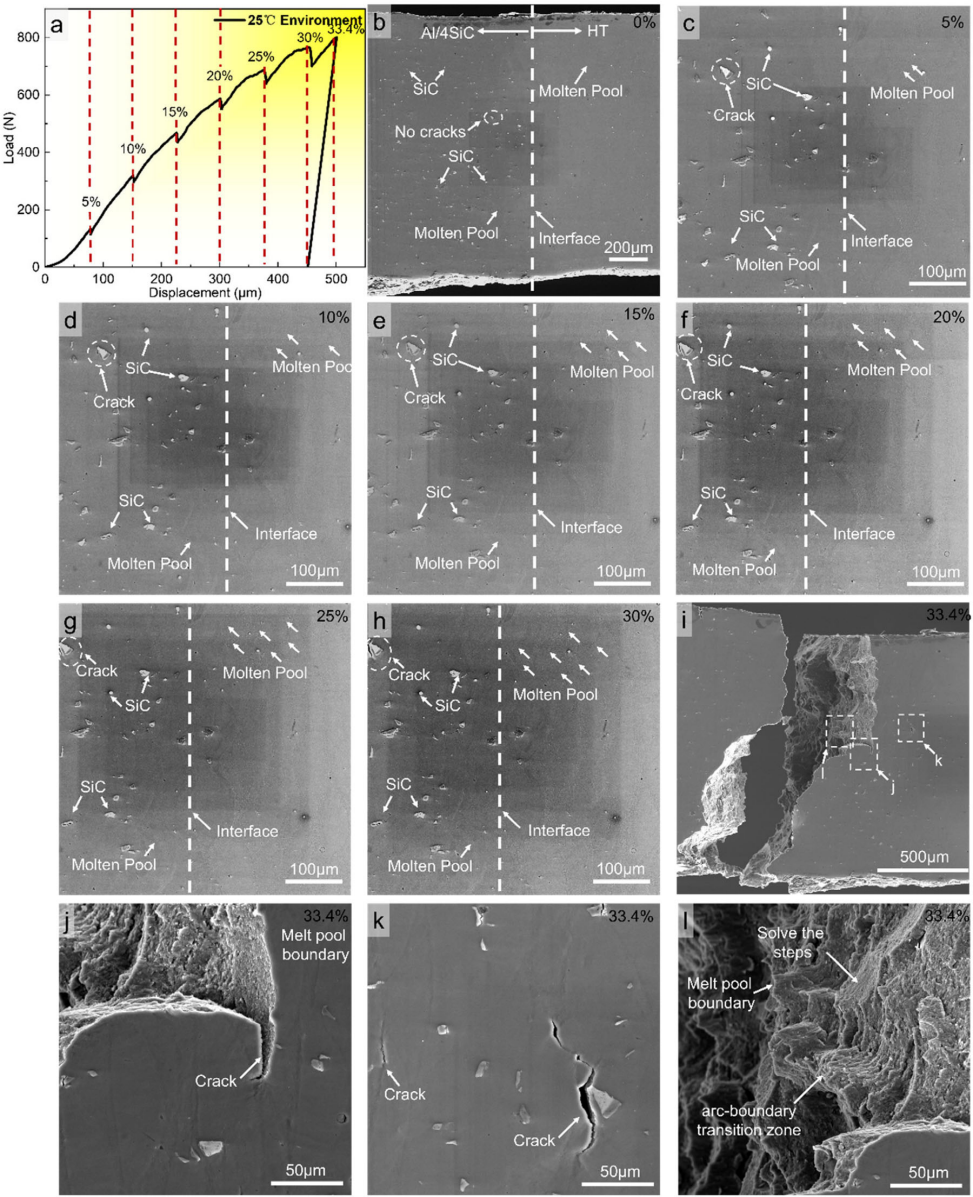
In situ SEM at 25°C revealing damage initiation and evolution in the HT‐4SiC bimetal. (a) Loading history annotated with interruption points for microstructural imaging. (b—h) Snapshots taken at increasing global strains (0%–30%). The HT (AlSiFeMnNiMg) side (right) and the Al/4SiC side (left) are separated by a sharp, pore‐free interface. (i) Low‐magnification view at 33.4% strain showing macroscopic crack paths arrested by microstructural heterogeneities. (j—l) Higher‐magnification details at ∼33.4% strain: (j) a crack arrested at the melt‐pool boundary, (k) crack coalescence within the Al/4SiC matrix away from the interface, and (l) step‐wise fracture along the arc‐boundary transition zone.

At 25°C, in situ tensile tests were conducted on interfacial specimens comprising AlSi10Mg reinforced with 4 vol% SiC and the AlSiFeMnNiMg alloy. Combined analysis of the load‐displacement response (Figure 4a) and SEM observations at successive strains (Figure 4b–i) enable a detailed interrogation of interfacial mechanics and micro‐damage evolution, with emphasis on the annotated regions and fracture features. At 0% strain (Figure 4b), the microstructures on both sides of the interface are intact. SiC particles are uniformly dispersed in the AlSi10Mg matrix with no cracks being observed, and the interface is tightly bonded, representing the initial and stable state. The melt‐pool architectures flanking the interface are complete, reflecting good as‐built quality on both sides. Upon loading to 5% strain (Figure 4c), microcracks first appear on the AlSi10Mg side. Owing to the elastic‐modulus mismatch between SiC and the matrix, stress concentrates around the particles, which act as damage nuclei. Although the melt‐pool boundaries on both sides begin to experience stress perturbations, their overall morphology remains largely undistorted. Between 10% and 30% strain (Figure 4d–h), cracks in the SiC/AlSi10Mg melt pools continue to extend. Two pathways are evident. First, crack nucleation along particle, matrix interfaces, followed by propagation through locally weaker interfacial regions. Second, crack penetration directly through the matrix, progressively undermines melt‐pool integrity. In this regime, SiC particles serve as persistent stress concentrators that repeatedly trigger crack initiation and coalescence within the melt pools. By contrast, the HT side, lacking rigid reinforcements, shows no pronounced cracking and deforms more compatibly, underscoring a fundamental asymmetry in deformation modes across the heterogeneous structure. With increasing strain, dislocations accumulate at melt‐pool boundaries on the AlSiFeMnNiMg side, reducing deformation compatibility and amplifying morphological distortion of the pools, as evidenced by their increasingly prominent outlines. Fracture occurs at 33.4% strain, with the failure located on the AlSi10Mg side (Figure 4i). Although the melt‐pool morphology on the AlSiFeMnNiMg side is more distorted, no cracks or microvoids are detected there. Higher‐magnification images of the fracture and adjacent region (Figure 4j–l) reveal a rippled fracture surface aligned with melt‐pool boundaries, indicating crack advance along these boundaries, together with signs of particle‐matrix decohesion (Figure 4k). These observations show that introducing SiC substantially elevates local stress concentration within the AlSi10Mg melt pools and severely diminishes plastic accommodation. Instead of being alleviated by sustained plastic flow, stresses promote particle‐assisted rapid crack growth, making this region the site of crack initiation and propagation. Conversely, the AlSiFeMnNiMg side does not act as a damage hot spot, highlighting the perturbative effect of SiC on melt‐pool deformation on the AlSi10Mg side [48]. Finally, the clear cleavage‐step morphology of the fracture surface indicates a brittle fracture mode along melt‐pool boundaries [49].

**FIGURE 5:**
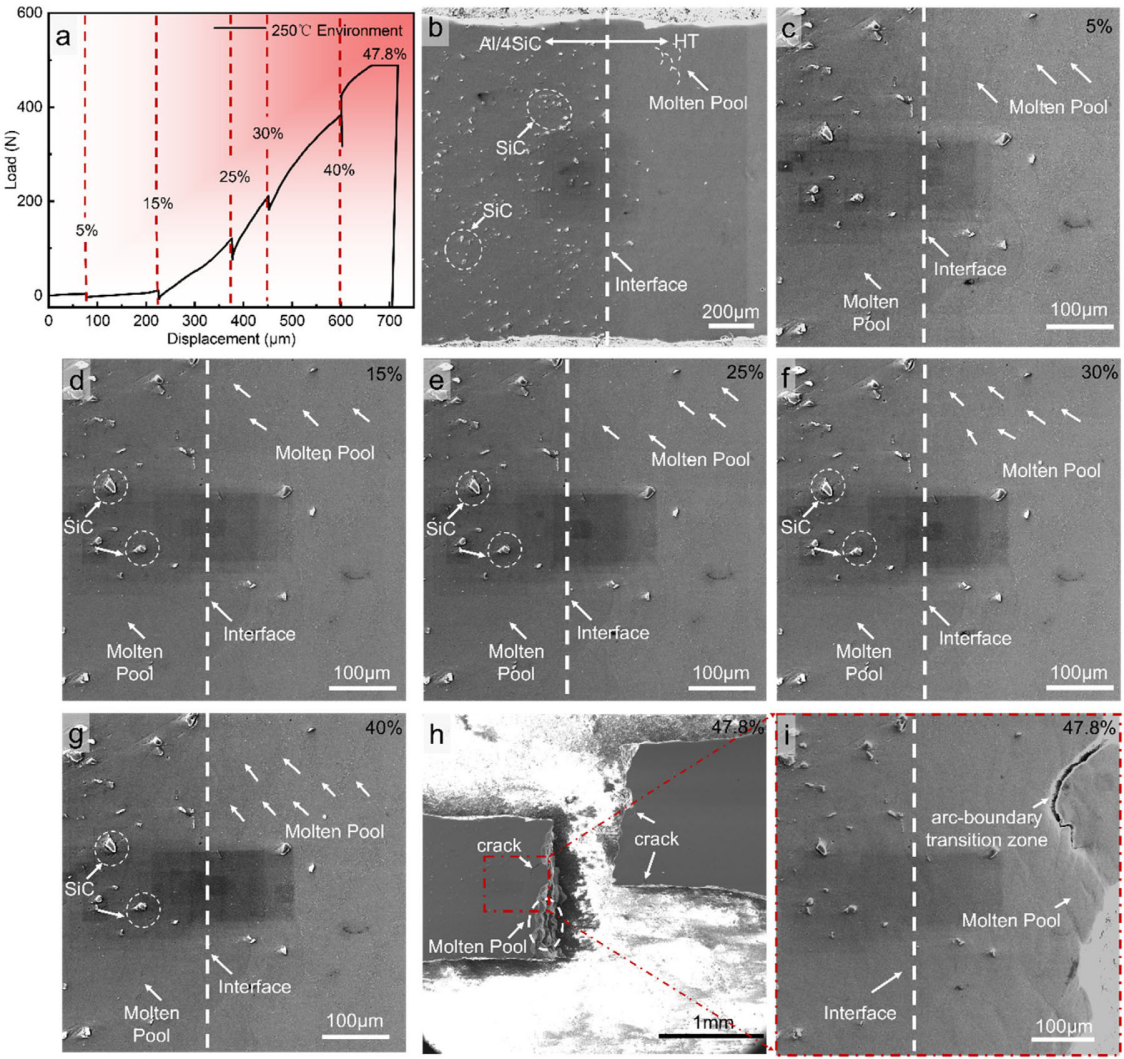
In situ SEM at 250°C elucidating high‐temperature damage evolution in the HT‐4SiC bimetal. (a) Loading history annotated with interruption points for microstructural imaging. (b—i) Snapshots taken at increasing global strains (0%–30%).

The in situ tensile deformation at elevated temperature (250°C) is shown in Figure 5. As displayed by the load‐displacement trace in Figure 5a, the step‐like features arise from stress relaxation during pauses and subsequent reloading. The black square patches correspond to surface carbonization induced by the electron beam. At the initial 0% strain (Figure 5b), discrete SiC particles are visible on the AlSi10Mg side (left), and a sharp interface with the AlSiFeMnNiMg alloy (right) is apparent. Both sides exhibit as‐printed melt‐pool morphology without discernible deformation features. At 5% strain (Figure 5c), subtle differences emerge near the interface with melt‐pool boundaries becoming slightly undulated, and incipient localized deformation appearing around SiC particles due to stress concentration. By 15% strain (Figure 5d), particle‐induced constraint strengthens on the AlSi10Mg side. Moreover, compositional and microstructural contrasts across the interface reduce deformation compatibility and amplify melt‐pool distortion. At 25% strain (Figure 5e), SiC particles act as strong obstacles to dislocation motion, promoting dislocation pile‐up and heightened local stresses. However, the interfacial deformation band broadens and the contrast in deformation between the two alloys becomes more pronounced, with cumulative strain increasingly accommodated between melt pools on the quinary alloy side. At 30% strain (Figure 5f), damage evolution accelerates in SiC‐depleted regions of AlSi10Mg the melt‐pool contours sharpen, and mechanical mismatch localizes strain at the interface region, positioning melt‐pool boundaries as potential damage pathways. At 40% strain (Figure 5g), microcracks nucleate around SiC particles and melt‐pool deformation is more evident on the quinary alloy side. Final fracture occurs at 47.8% strain (Figure 5h), notably within the AlSiFeMnNiMg alloy rather than at the interface. The fracture site exhibits a fan‐shaped protrusion, indicating separation along the melt‐pool arc. Higher‐magnification imaging near the fracture (Figure 5i) shows that cracks initiate at the transition along the melt‐pool arc boundary, serving as the earliest failure site. Columnar and dendritic structures formed during melt‐pool solidification render these boundaries microstructural “weak interfaces”. When a crack impinges on a melt‐pool boundary, grain orientation mismatch across the boundary converts the local stress state from uniaxial tension to a multiaxial, complex field, driving crack deflection, and branching. At elevated temperature, enhanced atomic mobility further accelerates grain‐boundary sliding and diffusion along the melt‐pool boundaries, promoting meandering crack advance that follows the melt‐pool contours and lengthens the fracture path.

### 2.4 FE Modeling and Validation

**FIGURE 6:**
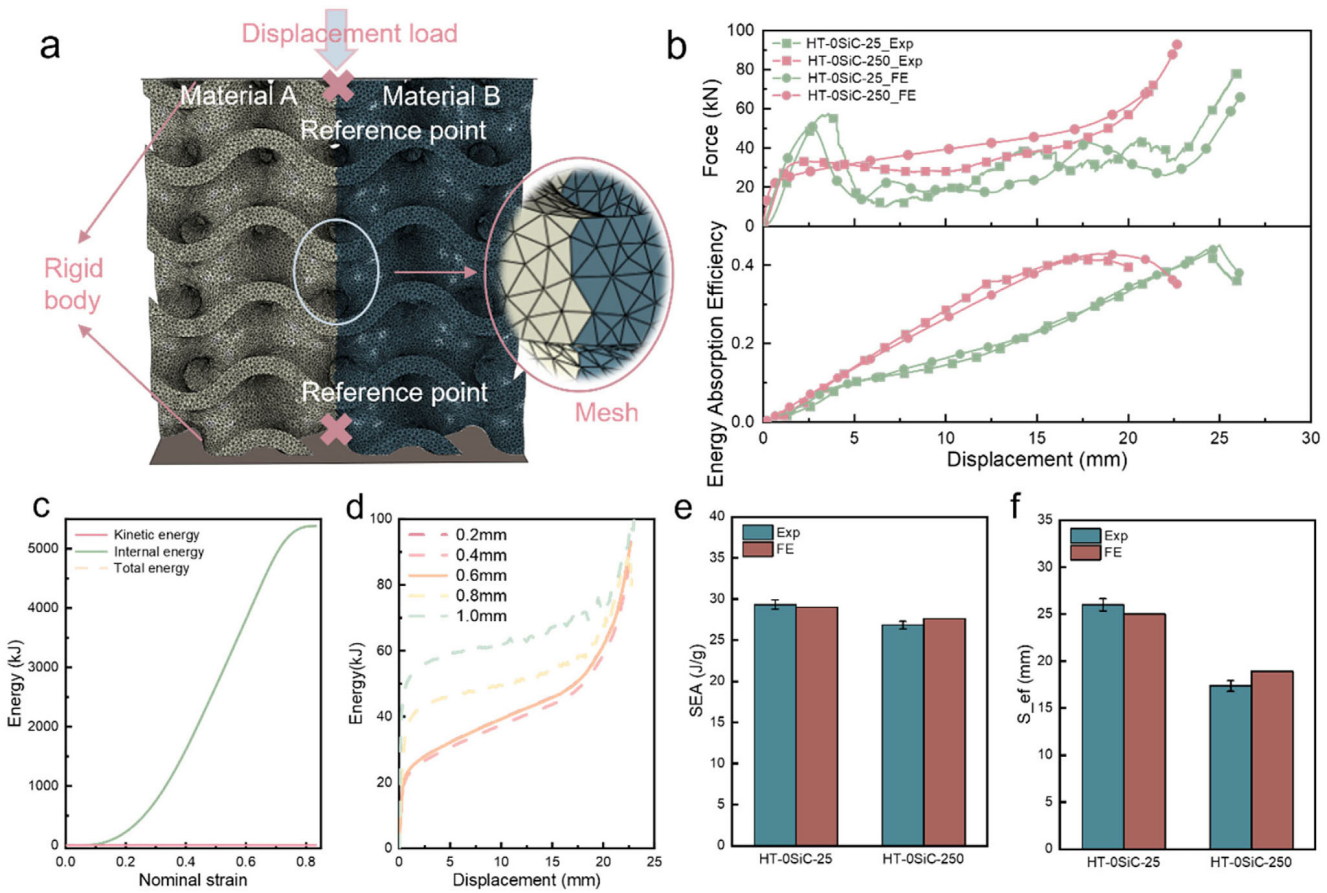
Finite‐element framework and validation for the bio‐inspired bimetallic TPMS. (a) FE model of bimetallic gyroid structures: rigid platen displacement loading, fixed base, material partition, and tetra mesh. Load and kinematics are extracted at the reference points. (b) Experiment‐simulation comparisons for load‐displacement of the HT‐0SiC TPMS at 25°C and 250°C. (c) Different types of energies during the FE quasi‐static loading. (d) Convergence results of the TPMS structure with different mesh sizes. (e) SEA and (f) S_ef_ from FE vs. experiment reveals accurate prediction of the densification onset.

Figure 6 systematically presents the compressive response of the basic materials, finite‐element (FE) modeling of the TPMS architecture, and stepwise validation. The engineering stress–strain curves under uniaxial compression for the monolithic and composite materials are shown in Figure S3. Relative to the AlSiFeMnNiMg matrix, the SiC‐containing composites exhibit a steeper initial slope, an earlier yield accompanied by a sharper stress rise, a markedly elevated peak stress, and a reduced ultimate strain, reflecting the familiar trade‐off whereby particle strengthening enhances load capacity at the expense of ductility. The FE model used for the bimetallic TPMS structures is shown in Figure 6a. Throughout compression, the ratio of kinetic to internal energy remained below 5% (Figure 6c), satisfying the criterion for quasi‐static loading [50]. As detailed in Figure 6d, we assessed six mesh sizes (1.0, 0.8, 0.6, 0.4, 0.2, and 0.1 mm). A 0.4‐mm mesh provided an optimal balance of efficiency, convergence, and fidelity in reproducing the lattice compression response and was therefore adopted for all subsequent analyses. To accommodate potential stress concentrations, we applied local mesh refinement around the small powder‐clearing apertures.

Materials parameters were imported into Abaqus via the material property interface. As seen in Figure 6e,f, numerical predictions agree well with experiments. For HT‐0SiC at both room temperature and 250°C, the simulated specific energy absorption (SEA) and S_ef_ deviate from measurements by <5%. This small discrepancy is consistent with prior reports and is attributable to unavoidable LPBF‐induced defects in test coupons, which particularly influence the elastic regime, whereas the FE model assumes ideal, defect‐free material [51, 52]. Overall, the close agreement between simulation and experiment substantiates the validity of our FE model.

### 2.5 Mechanical Responses and Fracture Behaviors of Bi‐Metal Structures

In this paper, specific energy absorption (SEA), mean crushing force (MCF), peak crushing force (PCF), crushing force efficiency (CFE), and plateau stress (σ_pl_) were selected as indicators to quantify the performance of the lattice structure [53, 54, 55].

First, calculate the energy absorption efficiency *f* of the structure on the load‐displacement curve. The calculation method is as follows:

(1) $$f=\frac{\int_{0}^{S}F\left(x\right)dx}{F_{\max}l_{0}}$$

In this equation, *S* denotes the compression displacement, *F(x)* represents the compression force, *F_max_* is the maximum compression force within the compression displacement interval [0, S], and *l_0_* is the initial height of the structure along the compression direction. The displacement corresponding to the highest point on the energy absorption efficiency curve is designated as *S_ef_*. This marks the transition from the platform stage to the dense stage and is considered the effective displacement. The ratio of *S_ef_* to *l_0_* is defined as the dense strain *ε_ef_*. The displacement at the conclusion of the elastic stage is designated as the yield displacement, *S_c0_*.

SEA is defined as the energy absorption per unit mass, calculated by dividing the total absorbed energy by the structural mass.

(2) $$\mathrm{SEA}=\frac{\int_{0}^{S_{\mathrm{ef}}}F\left(x\right)dx}{M}$$

In this equation, M denotes the mass of the structure.

The stress equation of the platform can be obtained from the load‐displacement curve.

(3) $$\sigma_{\mathrm{pl}}=\frac{1}{A_{0}}\cdot \frac{\int_{S_{c0}}^{S_{ef}}F(x)dx}{S_{ef}-S_{c0}}$$

MCF is defined as the ratio of energy absorption to effective displacement, mainly reflecting the load‐bearing capacity of the structure during the plastic deformation stage. It is calculated as follows:

(4) $$MCF=\frac{\int_{0}^{S_{ef}}F\left(x\right)dx}{S_{ef}}$$

PCF represents the maximum compression force within the compression displacement interval [0, *S_ef_*]. It has been demonstrated that the smaller the PCF, the smaller the load fluctuations during the compression process, and the more stable the load‐bearing performance of the structure. CFE is employed to delineate the load consistency of the structure during the compression process. The expression for CFE is as follows:

(5) $$CFE=\frac{MCF}{PCF}\times 100\%$$

**FIGURE 7:**
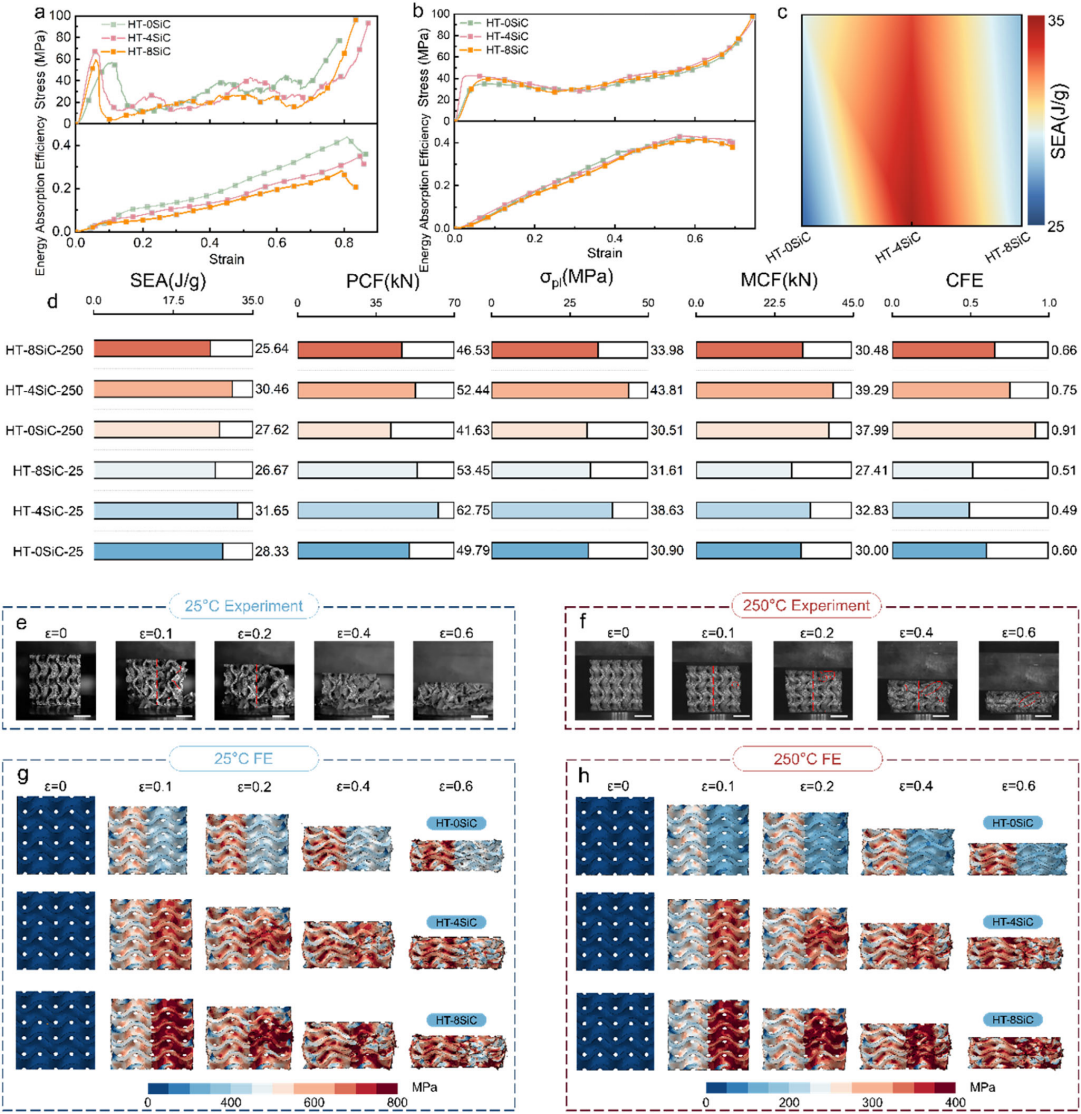
Temperature‐composition effects on compression and energy absorption of bio‐inspired bimetallic TPMS lattices, and correspondence with FE. (a) 25°C engineering stress–strain responses and accumulated energy per unit mass; all variants (HT‐0SiC, HT‐4SiC, and HT‐8SiC) show the canonical elastic‐plateau‐densification sequence, with increasing quasi‐linearly during the plateau. (b) At 250°C, plateaus become smoother and peak amplitudes converge, indicating thermally induced softening. (c) Specific energy absorption (SEA) map vs. temperature and SiC fraction highlights a pronounced “ridge” at 4 vol% SiC. (d) Summary metrics from experiments: SEA, plateau stress, mean crushing force (MCF), peak crushing force (PCF), and crushing‐force efficiency (CFE). (e—h) Deformation mechanism of the three types of TPMS structures.

Figure 7a,b summarize the compressive responses and energy‐absorption performance of the bimetallic TPMS lattices HT‐0SiC, HT‐4SiC, and HT‐8SiC at 25°C and 250°C. The data plotted are taken from the most representative specimens, showing the best repeatability and the smallest scatter, among five parallel tests. The curve features are consistent with the group‐wise trends and capture the core behavior. All statistical conclusions in this paper are drawn from the aggregate analysis of the full datasets, ensuring the reliability of the results. At 25°C, all three compositions exhibit the canonical three‐stage response, from linear elasticity to plateau platform to densification. The undulating plateau following the initial peak corresponds to cell‐by‐cell buckling and successive instabilities, while the energy‐absorption efficiency increases approximately linearly with strain. Upon heating to 250°C, the plateau becomes smoother and the peak amplitudes converge markedly, indicating that matrix thermal softening damps the intensity of local instabilities. Meanwhile, the response maintains a stable rise in the medium‐to‐high strain regime. In terms of composition, HT‐4SiC attains higher strength and plateau stress at both temperatures and, at high temperature, exhibits superior peak‐to‐mean consistency. Figure 7c further shows that within 25°C–250°C, 4 vol% SiC forms a performance ridge, sustaining high specific energy absorption across both temperatures. By contrast, the 0 vol% system benefits from thermal softening (higher CFE upon heating), whereas the 8 vol% system localizes more readily at high temperature due to aggravated modulus mismatch, leading to a weakened plateau. Overall, HT‐4SiC provides the most balanced combination of strength, energy absorption, and efficiency across temperatures, serving as the preferred composition for subsequent structural optimization and engineering applications.

As summarized in Figure 7d, the HT‐4SiC lattice provides the most favorable synergy between strength and energy absorption over the temperature range. At 25°C, its SEA reaches 31.65 J g^−^^1^ and the plateau stress rises to 38.63 mPa, which are 11.72% and 25.02% higher than HT‐0SiC and 18.67% and 22.21% higher than HT‐8SiC, respectively. When heated to 250°C, SEA and σ_pl_ of HT‐4SiC remain at 30.46 J g^−^^1^ and 43.81 mPa.Consistently, HT‐4SiC raises the peak and mean crushing forces by roughly 25.97% and 3.4% compared with HT‐0SiC, while maintaining a comparable crushing‐force efficiency. This performance stems from a moderate particle fraction that builds a stiff load‐bearing skeleton yet preserves sufficient matrix continuity, so that peak load, plateau level and deformation uniformity are improved simultaneously. At 250°C, matrix softening narrows the differences in peak and plateau levels, so the matrix‐dominated HT‐0SiC attains relatively high crushing‐force efficiency, although its SEA and σ_pl_ remain lower than those of HT‐4SiC. By contrast, the particle‐rich HT‐8SiC gains initial stiffness but its pronounced modulus mismatch accelerates shear‐band coalescence and block‐like crushing, which depresses SEA and σ_pl_ under both thermal conditions. Overall, a SiC fraction of 4 vol% maximizes energy dissipation and stable load carrying through efficient load transfer and a non‐percolating shear‐band network, in agreement with the load‐path analyses that show optimized stress partition at nodes and thin‐wall transitions [56].

Further, we have supplemented the results with comparative experimental plots illustrating the compressive mechanical performance of the HT‐4SiC bimetallic TPMS structure vs. the monolithic HT TPMS structure at both room temperature and elevated temperatures (Figure S4). As evident from the figures, the HT‐4SiC structure exhibits slightly superior energy absorption capabilities. Specifically, its Specific Energy Absorption (SEA) increased by 7.8% at room temperature and 13.49% at high temperature, respectively.

Simultaneously, we benchmarked our work against other metamaterials fabricated from common metals with similar porosity reported in the last two years (Figure S5) [57, 58, 59, 60, 61, 62, 63, 64, 65, 66, 67, 68]. By contrast, our Janus‐like bimetallic integrated design strategy successfully integrates the high‐temperature resistance of AlSiFeMnNiMg with the high strength provided by SiC‐reinforced AlSi10Mg into the emerging Gyroid TPMS architecture. This synergy enables the material to maintain a high, stable, and prolonged stress plateau during deformation. Consequently, our bimetallic metamaterials achieved unprecedented mechanical performance. The HT‐4SiC configuration reached SEA values exceeding 30 J/g at both room temperature and elevated temperatures. These values are significantly higher than those of other metamaterials made from monolithic aluminum alloys with comparable porosity.

Figure 7e–h depicts the deformation and failure modes of the three lattices, HT‐0SiC, HT‐4SiC, and HT‐8SiC, at 25°C (Figure 7e,f) and 250°C (Figure 7g,h), together with the corresponding evolution of von Mises stress fields obtained by finite‐element simulation. The modes are interpreted in conjunction with the stress–strain responses in Figure 7a,b. As shown in Figure 7e, specimens at 25°C undergo a multi‐stage sequence from linear elasticity to local buckling, layer‐by‐layer collapse, and final densification. In HT‐0SiC, buckling nucleates at the node. Thin‐wall transition zones and advances along 45° shear bands in a pronounced band‐like instability cascade. In HT‐4SiC, buckling onset is delayed and spatially more diffuse. Stress ripples on the plateau are markedly attenuated, indicating more uniform intercell load sharing. By contrast, HT‐8SiC exhibits intensified localization. The FE stress maps mirror the in situ observations, consistently identifying high‐curvature saddle points and necking regions of thin walls as stress‐concentration cores. Notably, the high‐stress regions in HT‐4SiC appear as discrete islands rather than long‐range connected bands, consistent with its smoother plateau and higher SEA. The continuous stress chains in HT‐8SiC rationalize its stronger localization tendency and earlier densification threshold. Upon heating to 250°C, HT‐0SiC exhibits reduced stiffness and peak load, yet diminished stress pulsations and a more uniform plateau, yielding improved crushing efficiency. HT‐4SiC maintains the most favorable plateau bearing and energy dissipation, whose stress fields alternate between nodes and webs in dotted‐band motifs that suppress early through‐thickness shear bands while preserving skeletal load capacity, resulting in robust performance retention from 25°C to 250°C. In HT‐8SiC, limited particle softening exacerbates modulus and thermal‐expansion mismatch with the matrix. High‐stress chains align along diagonals and accumulate at interfaces and nodes, producing rapidly spreading localization and a shortened plateau.

## 3 Conclusion

This work demonstrated a Janus‐like bio‐inspired, integrally 3D‐printed bimetallic structure that reconciled thermal stability with high load‐bearing capacity under service‐relevant temperatures. Using a customized dual‐hopper SLM route, a heat‐resistant AlSiFeMnNiMg alloy was co‐printed with AlSi10Mg‐xSiC (x = 0, 4, and 8 vol%) into TPMS, yielding compositionally partitioned interfaces with a narrow diffusion zone and no continuous porosity band. Multiscale characterization and in situ SEM at 25°C and 250°C identified a slender interfacial transition that was sensitive to early damage. When one side locally buckles or softens, the other side shunts and bridges the load, producing non‐percolating high‐stress clusters that suppress damage coalescence and delay crack formation and stabilize the plateau.

Thermo‐mechanical testing revealed a composition‐temperature optimum. Across testing temperatures, 4 vol% SiC maximizes energy‐absorption efficiency, delivering high specific energy absorption above 30 J g^−^^1^ and plateau stresses around 40 mPa, with the highest crushing‐force efficiency among all variants. Mechanistically, a moderate ceramic fraction constructed a stiff yet discontinuous load‐transfer network, promoting distributed shear‐banding and preventing stress percolation. By contrast, 0 vol% relied on matrix softening to raise crushing efficiency but offered lower absorption, whereas 8 vol% suffered from modulus and thermal‐expansion mismatch that accelerated early localization and blocky crushing. Finite‐element models reproduced the measured stress–strain response, thereby validating the load‐shunting mechanism.

These findings consolidated actionable design rules for bimetallic structures by tuning SiC volume fraction to maximize load shunting without enabling stress percolation, with approximately 4 vol% as a robust default for strength‐absorption synergy. The approach was readily extensible to other bimetallic structures fabricated by ceramic reinforced alloy, offering a scalable pathway to lightweight thermal‐protective and load‐bearing lattices for spacecraft and satellite systems.

## 4 Methods

### 4.1 Fabrication of Bi‐Metal Structures

Gas‐atomized AlSiFeMnNiMg powder and spherical AlSi10Mg powder premixed with SiC particles at designed volume fractions x = 0, 4, and 8 vol% served as the feedstocks. Prior to blending, AlSi10Mg was dried at 55°C for 6 h and SiC at 165°C for 6 h. The dried powders were then mixed in a tumbler mixer (HL1‐5/10, Jun‐Long Technology, China) at 300 rpm for 4 h to promote uniform dispersion of SiC. The nominal chemical compositions of AlSiFeMnNiMg and AlSi10Mg‐xSiC are listed in Table S1. All lattice specimens were produced by selective laser melting (SLM) on an EOS M290 system under nitrogen protection, maintaining the residual oxygen below 1% throughout the build. Printing and compensation parameters for the two material systems were separately optimized to ensure dimensional accuracy and consistent density; the final processing windows are summarized in Table S2.

### 4.2 Morphological and Interfacial Characterization

#### 4.2.1 Quality Inspection

Relative density was determined by the Archimedes method; each specimen was measured five times and the average was reported. Pore evolution in the three bimetal architectures was assessed by X‐ray micro‐computed tomography (ZEISS Xradia 620 Versa). Reconstructed volumes were segmented using a global gray‐level threshold to distinguish voids from the consolidated alloy, enabling quantitative mapping of pore distribution.

#### 4.2.2 Microstructure Characterization

Powder morphology and chemistry were characterized by field‐emission SEM (FE‐SEM) coupled with energy‐dispersive X‐ray spectroscopy (EDS). Metallographic specimens were ground sequentially with 240–5000 grit SiC papers and then mirror‐polished using 3.5 and 1.5 µm diamond pastes. Local elemental quantification was obtained by a field‐emission electron probe microanalyzer (JEOL JXA‐iHP200F). Initial grain orientation and texture were mapped by electron backscatter diffraction (EBSD) on a Verios 5 UC (Thermo Fisher).

### 4.3 Mechanical Experiments

#### 4.3.1 In Situ Tensile Tests

Tensile experiments were performed in an in situ manner at 25°C and 250°C using miniaturized specimens with gauge dimensions of 1.5 × 1.5 × 0.7 mm^3^. For elevated‐temperature tests, samples were heated at 25°C min^−^^1^ to the set point and held 10 min to reach thermal equilibrium before loading. The tensile stage was mounted inside a field‐emission SEM equipped with a micro‐tension module, enabling synchronous high‐resolution imaging during deformation. An interrupted‐loading protocol was adopted to document microstructural evolution. Observations were taken at crosshead displacements of 0, 75, 225, 300, 450, and 600 µm, corresponding to nominal strains of 0%, 5%, 15%, 25%, 30%, and 40%, respectively. At each stop, the crosshead was briefly paused to acquire images accompanied by a small, transient load drop). After each imaging interval, the tensile test was resumed and the same strain rate was utilized.

#### 4.3.2 Quasi‐Static Compressive Tests

The basic compressive tests on four materials, including AlSiFeMnNiMg, Al/0SiC, Al/4SiC, and Al/8SiC, were conducted using an uniaxial testing machine (Instron 8802) under quasi‐static loading conditions. Specimens were mounted with precise alignment to ensure uniform loading along the axis. The loading velocity was set to 2  mm/min in accordance with ASTM E209, with force‐displacement data continuously recorded throughout the tests [69]. Each test group comprised five standard specimens, which were manufactured using SLM for testing basic mechanical properties. After that, a total of 6 groups of quasi‐static compression tests were conducted on three types of TPMS specimens (HT‐0SiC, HT‐4SiC, and HT‐8SiC) under different environmental temperatures (25°C and 250°C), with five specimens in each group. The testing device and actual loading were illustrated in Figure S6. High‐resolution cameras were employed to capture images of the deformation process. High‐temperature tests were carried out inside a 500°C environmental chamber, at a loading rate of 1 mm/min. The loading process was sampled and recorded at 10 Hz, capturing the quasi‐static compression process for structural failure mode analysis.

### 4.4 Numerical Simulations

Quasi‐static compression simulations were performed in ABAQUS/Explicit 2020. The lower platen was fully constrained, while the upper platen was restricted to move only along the global *z*‐axis. To reduce computational cost, lattice sheets were modeled with first‐order, three‐node triangular shell elements (S3R), and the two platens were modeled as discrete rigid bodies (R3D4). A general contact algorithm was used with “hard” normal behavior and a tangential Coulomb friction coefficient of 0.3. The shell thickness of the gyroid (G‐type) struts was set to 0.85 mm so that the FE models matched the relative density of the printed specimens. A nominal compressive engineering strain of 0.80 was applied to the structure. Consequently, the Johnson‐Cook (J‐C) constitutive model was employed to define the constitutive relationship of different alloys [70]. Specifically, the alloy can be approximated as a linear hardening elastoplastic material, simplifying the J‐C constitutive equation to:

(6) $$\sigma =\left(A+B\varepsilon_{e}^{n}\right)\left(1+C\ln\frac{\dot{\varepsilon }}{{\dot{\varepsilon }}_{0}}\right)\left(1-{\left[\frac{T-T_{room}}{T_{m}-T_{room}}\right]}^{m}\right)$$

where A is the initial yield stress, B is the hardening coefficient, C is the strain rate coefficient, n is the strain hardening exponent, and m is the thermal softening exponent, *ε_e_* is the effective plastic strain, and *ε* = *ε*_0_ is the normalized equivalent plastic strain rate. T, T_m_, and T_room_ are the temperature of the material, the melting temperature, and room temperature, respectively. To capture the failure behavior of the structures, the J‐C damage model was used. The J‐C constitutive parameters of the four alloys, obtained through experiments and least‐squares fitting, are listed in Table S3. The model is based on real geometry with the mesh convergence for independence testing. The final element size ensures that the buckling waveform can be analyzed and the computational complexity is manageable.

## Author Contributions

Conceptualization: Z.D., W.C., Y.H., B.J., and H.H., Methodology: Z.D., W.C., Y.H., B.J., and H.H., Investigation: Z.D., W.C., and Y.H., Visualization: Z.D., W.C., Y.H., and B.J., Supervision: Z.D., X.W, and H.H., Writing – original draft: Z.D., W.C., and Y.H., Writing – review & editing: Z.D., B.J., X.W, and H.H.

## Conflicts of Interest

The authors declare no conflicts of interest.

## Supporting information




**Supporting File**: advs74179‐sup‐0001‐SuppMat.docx.

## Data Availability

The data that support the findings of this study are available from the corresponding author upon reasonable request.
