# Supplementary material for: A Janus‐Like Bio‐Inspired Strategy for 3D‐Printed Bimetallic Metamaterials with Excellent Thermal‐Protection and Load Bearing Capacity
Source: Adv Sci (Weinh). 2026 Feb 5;13(20):e24116. doi: 10.1002/advs.202524116 (PMC13067764; doi:10.1002/advs.202524116)
Supplement: Supplementary file 1 — Supporting File: advs74179‐sup‐0001‐SuppMat.docx. [file ADVS-13-e24116-s001.docx]

Supplementary Materials for

**A Janus-like bio-inspired strategy for 3D-printed bimetallic metamaterials with excellent thermal-protection and load bearing capacity**

*Zhicheng Dong, Wei Cheng, Yu He, Ben Jia, Xiaopeng Wan, Heyuan Huang**

*Corresponding author Email: huangheyuan@nwpu.edu.cn

**This PDF file includes:**

Figs. S1 to S6

Tables S1 to S3


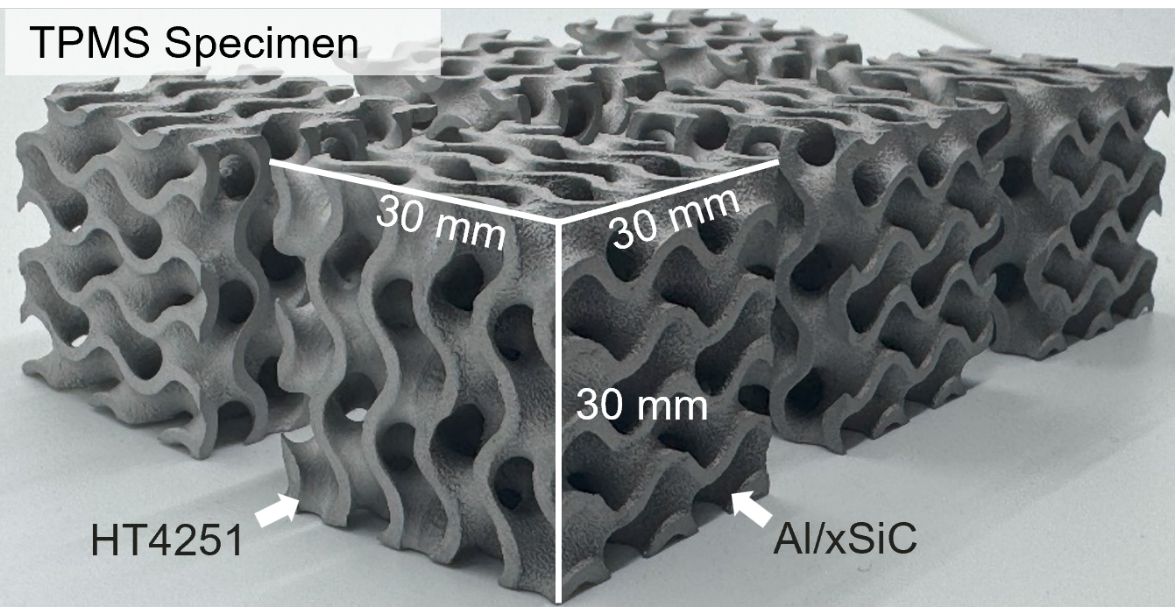


Fig. S1.

The size of SLM-printed bimetallic TPMS structures.


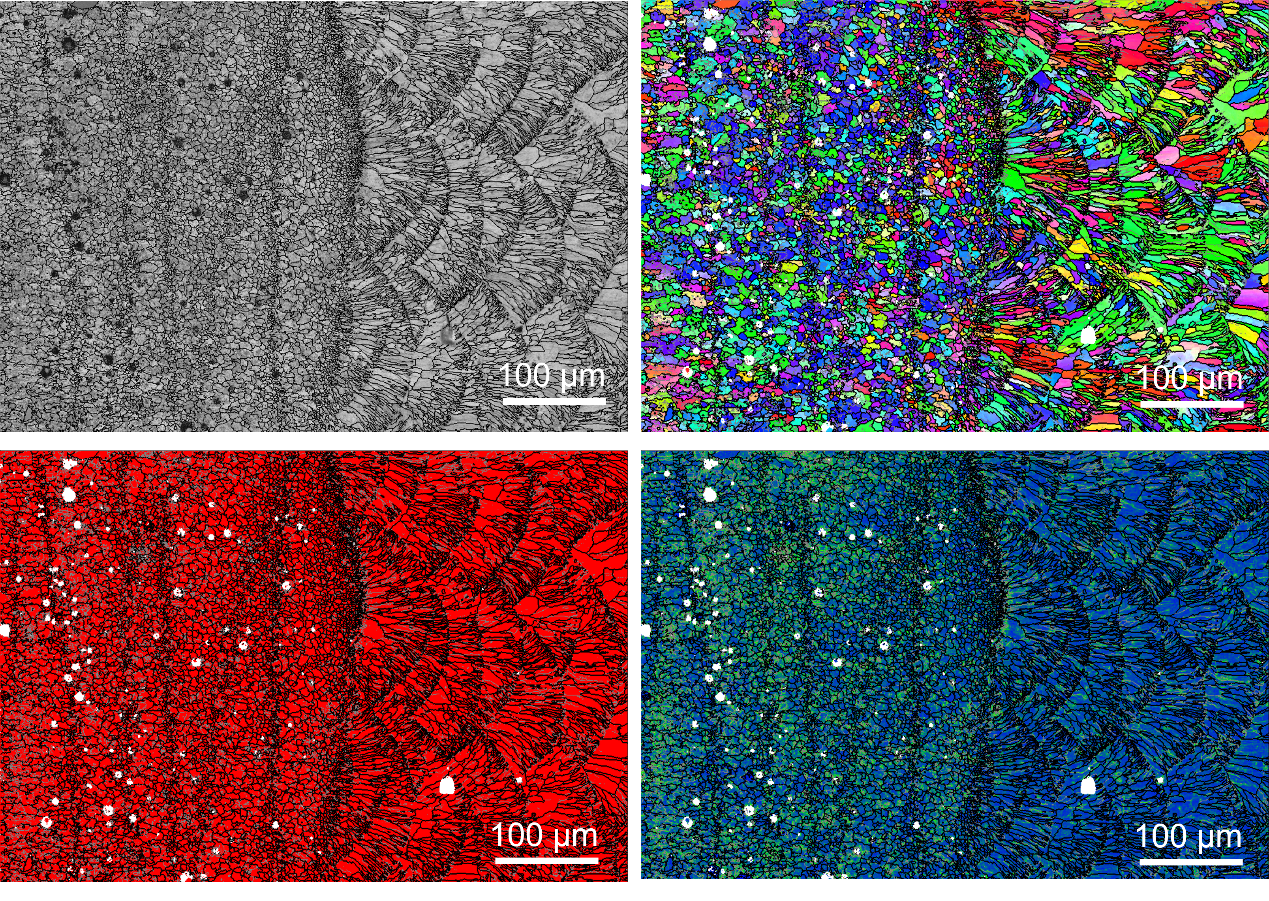


Fig. S2.

Initial EBSD map of the HT-4SiC in-situ tensile specimen.


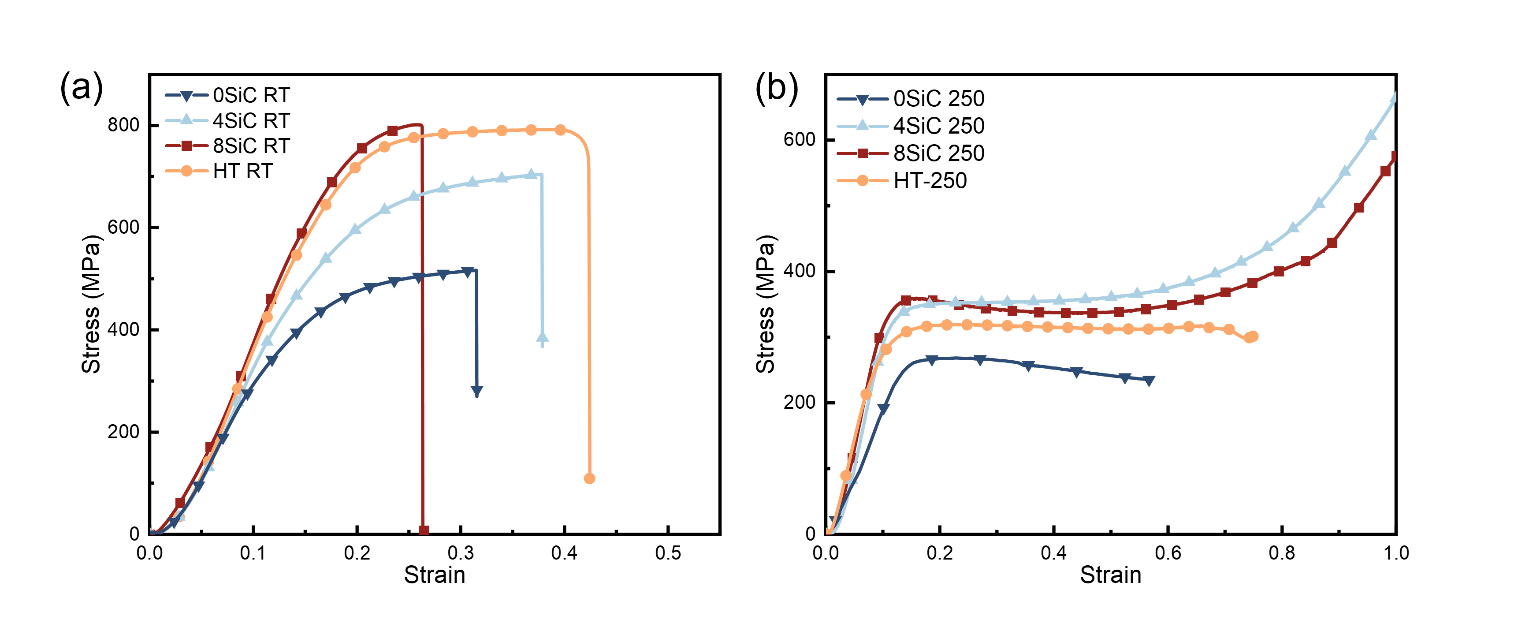


Fig. S3.

**a** Quasi-static compression of the four materials at 25℃ used to calibrate the elastic-plastic constitutive laws (HT: AlSiFeMnNiMg; Al/xSiC composite with x = 0, 4, 8 vol%). **b** Quasi-static compression of the four materials at 250℃.


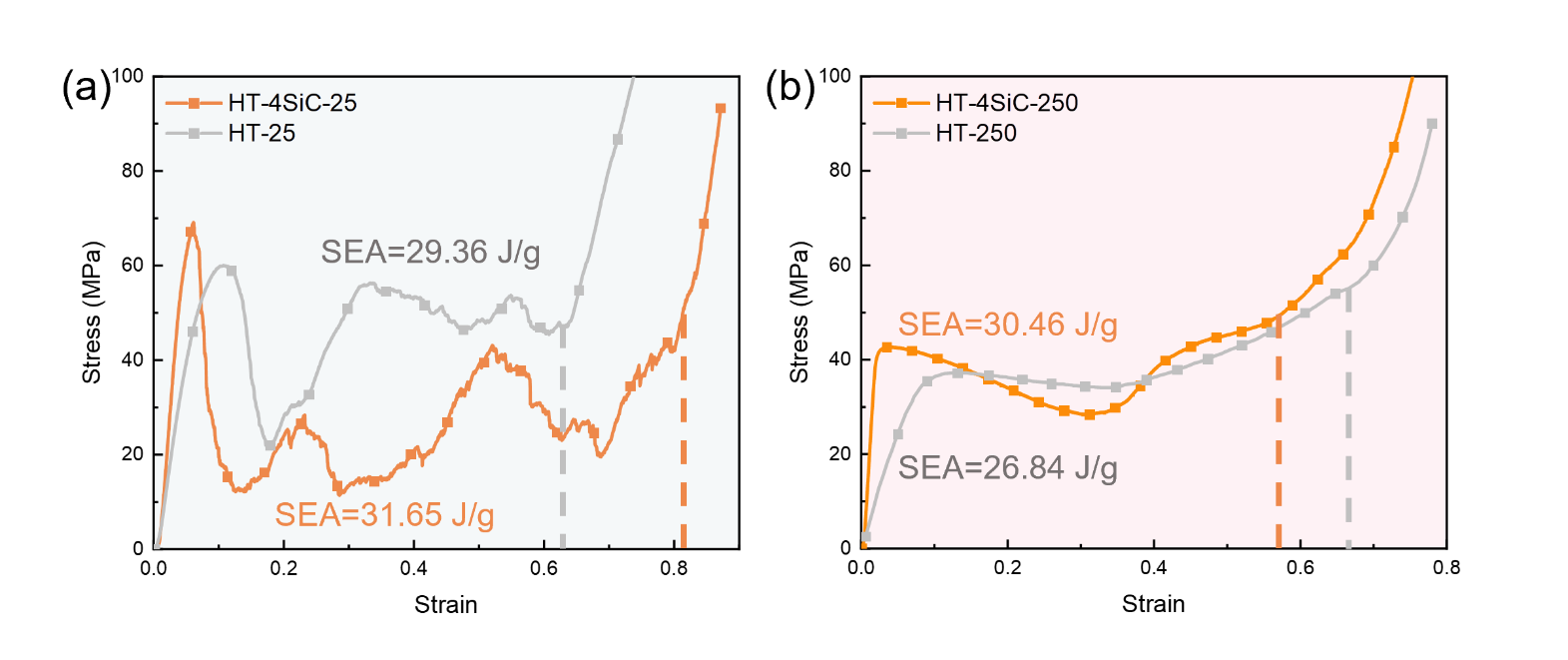


Fig. S4.

The comparison between HT-4SiC and pure HT lattices: (a) 25℃, (b) 250℃.


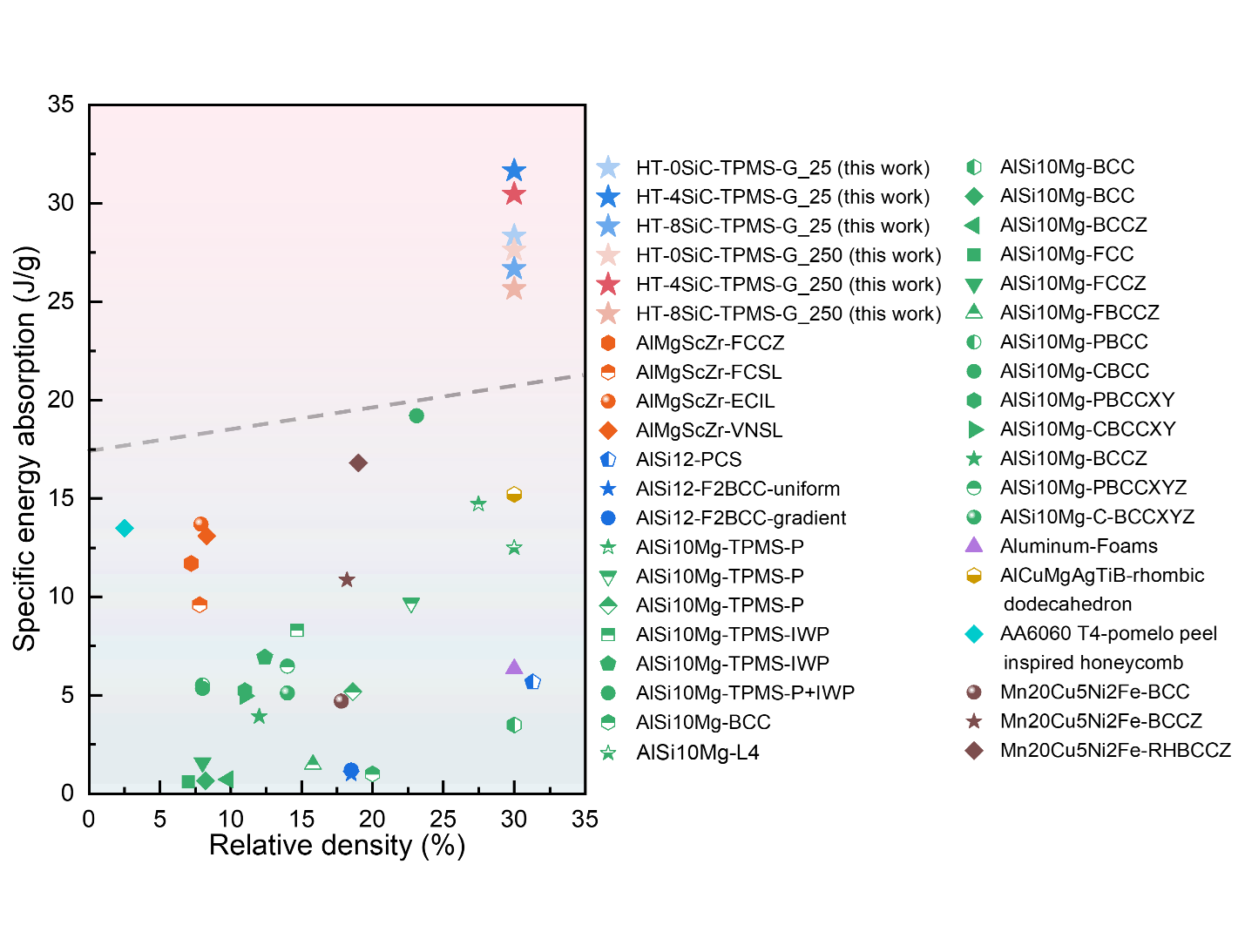


Fig. S5.

Comparison of the SEA of our HT-xSiC metamaterials with previously reported metallic metamaterials


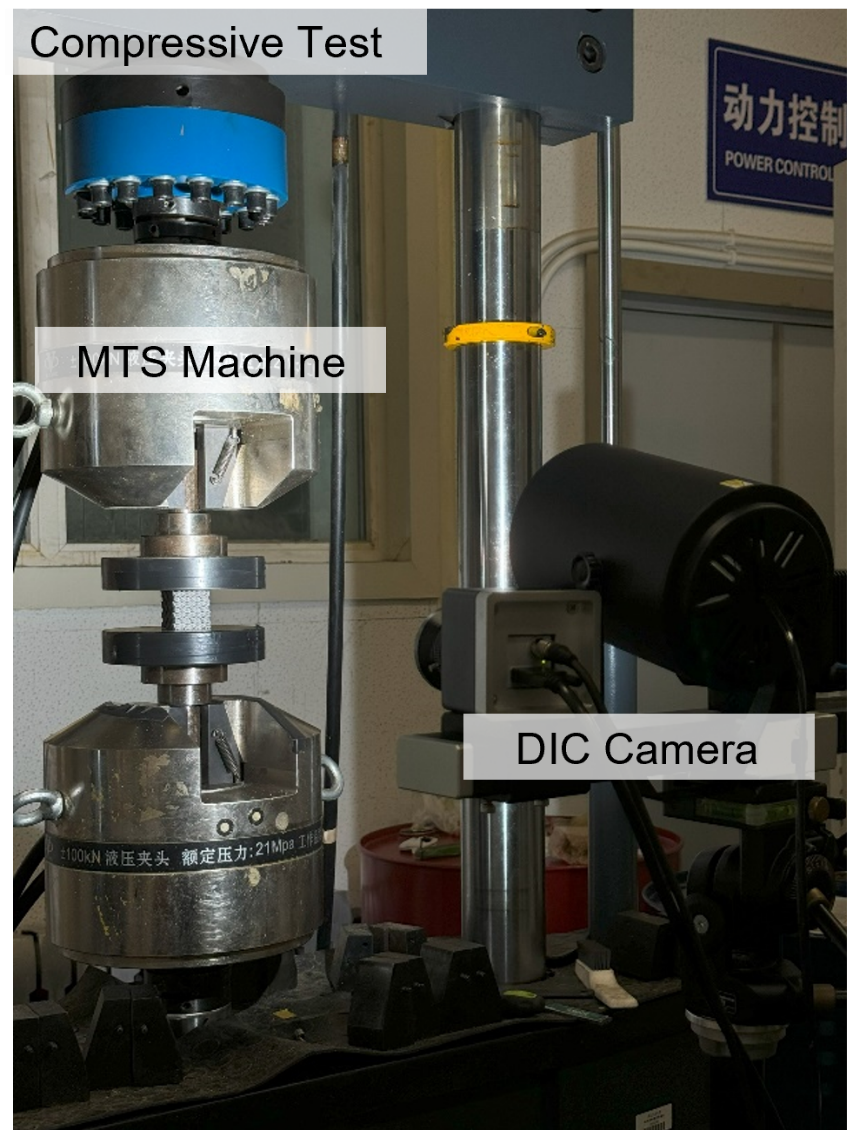


Fig. S6.

Quasi-static compressive test set-up

Table S1.

The chemical composition of AlSiFeMnNiMg alloy and AlSi10Mg-xSiC powder (wt.%)

|  | *Si* | *Fe* | *Ni* | *Mn* | *Mg* | *C* | *Al* |
| --- | --- | --- | --- | --- | --- | --- | --- |
| *AlSiFeMnNiMg* | *7.8* | *2.3* | *1.4* | *1.2* | *0.2* | */* | *Bal.* |
| *AlSi10Mg-0SiC* | *12.16* | */* | */* | */* | *0.42* | */* | *Bal.* |
| *AlSi10Mg-4SiC* | *11.69* | */* | */* | */* | *0.32* | *6.12* | *Bal.* |
| *AlSi10Mg-8SiC* | *17.39* | */* | */* | */* | *0.27* | *11.93* | *Bal.* |

Table S2.

SLM process parameters used for different materials

| *Printing parameters* | *Values* | | | |
| --- | --- | --- | --- | --- |
|  | *HT* | *0SiC* | *4SiC* | *8SiC* |
| *Laser Power (W)* | *385* | *385* | *305* | *250* |
| *Scanning speed (mm/s)* | *1600* | *1600* | *1500* | *1500* |
| *Scanning spacing (μm)* | *0.15* | *0.15* | *0.12* | *0.08* |
| *Layer thickness (μm)* | *0.03* | *0.03* | *0.03* | *0.03* |
| *Spot diameter (μm)* | *71* | *71* | *71* | *71* |
| *Spot compensation (μm)* | *-0.16* | *-0.16* | *-0.17* | *-0.17* |

Table S3.

J-C constitutive parameters of different alloys

|  | *A* | *B* | *C* | *m* | *n* | *T_r_* | *T_m_* |
| --- | --- | --- | --- | --- | --- | --- | --- |
| *AlSiFeMnNiMg* | *552* | *736* | *0.017* | *1.68* | *0.64* | *25* | *790* |
| *AlSi10Mg-0SiC* | *211* | *230* | *0.015* | *0.86* | *0.42* | *25* | *540* |
| *AlSi10Mg-4SiC* | *250* | *136* | *0.025* | *1.50* | *0.5* | *25* | *540* |
| *AlSi10Mg-8SiC* | *300* | *471* | *0.009* | *3.95* | *0.3* | *25* | *540* |
